# Social relationship dynamics mediate climate impacts on income inequality: evidence from the Mexican Humboldt squid fishery

**DOI:** 10.1007/s10113-021-01747-5

**Published:** 2021-03-24

**Authors:** Laura G. Elsler, Timothy Haight Frawley, Gregory L. Britten, Larry B. Crowder, Timothy C. DuBois, Sonja Radosavljevic, William F. Gilly, Anne-Sophie Crépin, Maja Schlüter

**Affiliations:** 1 Stockholm Resilience Centre, Stockholm University, Kräftriket 2B, 11419 Stockholm, Sweden; 2 Hopkins Marine Station, Stanford University, 120 Ocean View Blvd, Pacific Grove, CA 93950 USA; 3 Program in Atmospheres, Oceans, and Climate, Massachusetts Institute of Technology, Bldg. 54, 77 Massachusetts Avenue, Cambridge, MA 02139 USA; 4 Stockholm University, Stockholm Resilience Centre, 11419 Stockholm, Sweden; 5 The Beijer Institute, The Royal Swedish Academy of Sciences, 10405 Stockholm, Sweden

**Keywords:** Social structures, Environmental changes, Social-ecological systems modeling, Inequality, Humboldt squid fishery

## Abstract

Small-scale fisheries are critically important for livelihoods around the world, particularly in tropical regions. However, climate variability and anthropogenic climate change may seriously impact small-scale fisheries by altering the abundance and distribution of target species. Social relationships between fishery users, such as fish traders, can determine how each individual responds and is affected by changes in fisheries. These informal cooperative and competitive relationships provide access, support, and incentives for fishing and affect the distribution of benefits. Yet, individuals’ actions and impacts on individuals are often the primary focus of the economic analyses informing small-scale fisheries’ formal management. This focus dismisses relevant social relationships. We argue that this leads to a disconnect between reality and its model representation used in formal management, which may reduce formal fisheries management’s efficiency and efficacy and potentially trigger adverse consequences. Here, we examine this argument by comparing the predictions of a simple bioeconomic fishery model with those of a social-ecological model that incorporates the dynamics of cooperative relationships between fish traders. We illustrate model outcomes using an empirical case study in the Mexican Humboldt squid fishery. We find that (1) the social-ecological model with relationship dynamics substantially improves accuracy in predicting observed fishery variables to the simple bioeconomic model. (2) Income inequality outcomes are associated with changes in cooperative trade relationships. When environmental temperature is included in the model as a driver of species production dynamics, we find that climate-driven temperature variability drives a decline in catch that, in turn, reduce fishers’ income. We observe an offset of this loss in income by including cooperative relationships between fish traders (oligopoly) in the model. These relationships break down following species distribution changes and result in an increase in prices fishers receive. Finally, (3) our social-ecological model simulations show that the current fishery development program, which seeks to increase fishers’ income through an increase in domestic market demand, is supported by predictions from the simple bioeconomic model, may increase income inequality between fishers and traders. Our findings highlight the real and urgent need to re-think fisheries management models in the context of small-scale fisheries and climate change worldwide to encompass social relationship dynamics.

**Supplementary information:**

The online version contains supplementary material available at (10.1007/s10113-021-01747-5)

## Introduction

Climate change and variability trigger changes in the abundance and distribution of target species, influencing fishers, fish traders, and their relationships (Cinner et al. 2012; Pinsky and Mantua 2014; Britten et al. 2016; Elsler 2020). Regional fisheries’ productivity may shift by up to 50% (Cheung et al. 2010), and species distributions is likely to change in response to climate change (Pinsky et al. 2013). Climate-driven redistribution of fisheries’ maximum catch potential is expected to be most severe across tropical regions (Cheung et al. 2010), where informal cooperative and competitive relationships between and amongst fishers and traders govern small-scale fisheries (Drury O’Neill et al. 2018; Ferrol-Schulte et al. 2014). Changes in species’ dynamics can affect fishers’ livelihoods (Le Bris et al. 2018), including cases where they alter social relationships that govern benefit distribution (Adger 1999; Drury O’Neill et al. 2018). Effective fisheries management must then develop an understanding of social relationship responses to climate change and their consequences for managing the distribution of fisheries’ benefits (Adger 1999). One recent strand of empirical fisheries research has thus focused on the impacts of climate change on fishers (Pinsky and Mantua 2014; Rogers et al. 2019) and the propagation of those impacts to other users such as traders (Fleming et al. 2014).

In small-scale fisheries, social relationships can mediate outcomes for local economies and welfare. Relationships between traders can help create economic buffers for fishers; for instance, traders might buy fish that they did not demand but can sell it to another trader (Gonzalez-Mon et al. 2019). Fishers’ higher flexibility to catch what is available can increase their income. However, social relations may also negatively affect welfare outcomes. Competition and dependence in relationships can help determine how social relationships affect welfare outcomes. Empirical evidence shows that traders can act as gatekeepers to global markets (Ferse et al. 2014; Elsler et al. 2019), provide fishing permits, equipment, or loans (Frawley et al. 2019c; Basurto et al. 2013). These interactions can create dependencies and reduce fishers’ ability to negotiate competitive prices (Ferrol-Schulte et al. 2014; Basurto et al. 2020). Also, cooperation amongst traders can reduce market competition and enables them to fix the prices at which they buy fish below market value (Wamukota et al. 2014). Low prices paid by traders, in turn, can lead to low fishers’ income. Thus, social relationships can be important determinants of welfare outcomes in fisheries.

Prominent empirical evidence highlights the need to incorporate climate change impacts on social relationships to achieve effective fisheries management (Adger 1999; Daw et al. 2009). Nonetheless, their inclusion remains rare in the predictive bioeconomic models widely used for management support (Clark 1985; Nielsen et al. 2018; Prellezo et al. 2012; Naufal et al. 2019; Cabral et al. 2018; Fryxell et al. 2017). Recent advances in bioeconomic models involve assessing equity in social outcomes (Hutton et al. 2016; Plaganyi et al. 2013) and fisheries interactions with actors in the seafood supply chain (Christensen et al. 2011; Kaplan and Leonard 2012). These models include static representations of social relationships. In contrast, social-ecological models are a new class of models that explicitly represent the importance of social dynamics and relationships in response to and as drivers of ecological dynamics (Schlüter et al. 2012; Lade et al. 2015).

We argue that social relationships are relevant to managing fisheries effectively. Analogous to social relationships, such relevance has been demonstrated for ecological relationships. Ecosystem-based management scholars have shown that omitting the effect of environmental changes on ecological relationships, such as those between predator and prey, can lead to misguided assessments of fisheries (McLeod and Leslie 2009). Omission of relevant social relationships may lead to similar biases. Climate change can affect cooperative and competitive relationships between fishery users (Lindkvist et al. 2017). Yet, understanding and predicting the impacts of relationship dynamics on fishery outcomes remain a major scientific gap. Social-ecological models, particularly agent-based models, have highlighted an important role for social relationship dynamics, such as cooperation and competition between fishers, cooperatives, and fish traders (BenDor et al. 2009; Lindkvist et al. 2017). Therefore, social-ecological modeling approaches may be useful to bridge the gap between existing bioeconomic models and the reality in which social relationship dynamics shape small-scale fishery outcomes.

This paper investigates how fishery users’ relationships can mediate the impacts of climate change (henceforth, including climate variability changes) on the distribution of fishery benefits. We chose the Humboldt squid fishery because of its significant and well-documented response to recent changes in environmental and climate trajectories (Frawley et al. 2019a) and can serve to motivate a reappraisal of small-scale fisheries management models worldwide. We compare our model to a simple bioeconomic model currently used for fisheries management. Then, we test both models’ respective ability to predict observed fishery variables. Finally, we use the models to analyze income and income inequality in the squid fishery for different fisheries development programs and climate change trajectories. Thus, our analysis integrates social relationship dynamics in predictive social-ecological models to support effective small-scale fisheries management through these steps.

## Social-ecological dynamics in the Mexican Humboldt squid fishery

The Guaymas basin is the geographic center of the Gulf of California. It represents a biologically productive oceanographic transition zone where the ecosystem is sensitive to changes in water temperature (Lluch-Belda et al. 2003). Climate change in the Gulf of California is strongly influenced by El Niño and La Niña oscillations and an increasing temperature trend (Petatan-Ramirez et al. 2019) due to anthropogenic forcing. These atmospheric and oceanographic drivers affecting mesoscale circulation patterns, upwelling, and primary productivity are often assessed using remotely sensed proxies like sea surface temperature variability (SST) (Lluch-Cota et al. 2010; Robinson et al. 2016; Frawley et al. 2019a).

**Fig. 1:**
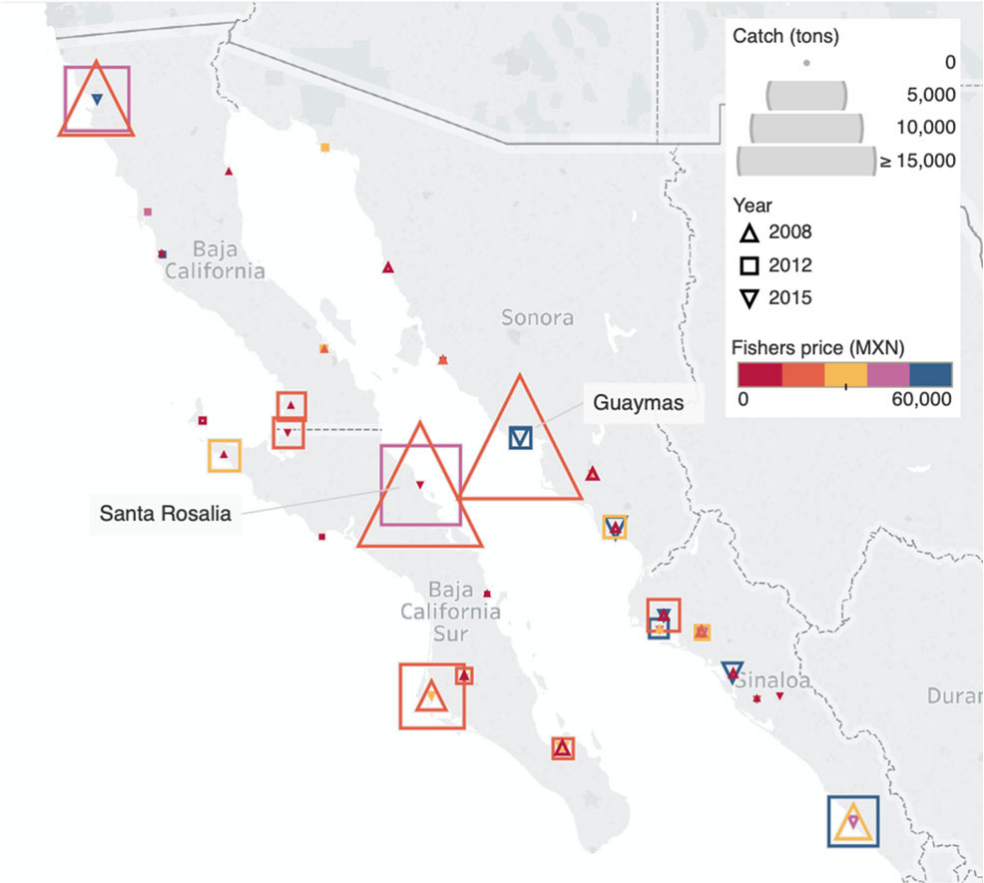
The map illustrates three years with different climatic conditions of Humboldt squid catches and fishers’ prices registered by fishery reporting office. The markers’ size represents catch volumes (tons); colors indicate ranges of fishers’ pricesin Mexican pesos (MXN) per ton. Santa Rosalìa and Guaymas constitute the core fishing centers

The Humboldt squid is a migratory, opportunistic predator which forages in highly productive regions like the Guaymas basin (Hoving et al. 2013). It has a very flexible life history strategy and can change its phenotypic life span and body size to accommodate variable environmental and oceanographic conditions (Hoving et al. 2013). Though the core fishing grounds of the Mexican commercial squid fishery are offshore of the ports of Guaymas and Santa Rosalìa (Fig. 1), episodic landings have also been recorded in the Northern Gulf and along the Pacific coast. Historical catch fluctuations on either side of the Baja California peninsula and the recent collapse of the fishery within the Gulf have been reported to correlate with anomalous climatic and oceanographic conditions (Hoving et al. 2013; Frawley et al. 2019b).

Responding to changes in squid catch volumes and migration patterns requires flexible and dynamic interactions between fishers and traders. The squid fishery in Santa Rosalìa and Guaymas started commercially in the 1980s, primarily to supply Asian markets (Cruz-González et al. 2011). Our analysis focuses on small-scale fishers, who landed the majority of squid after the 1990s (Cruz-González et al. 2011). This jig-based fishery allows opportunistic fishing across Northwest Mexico. For instance, high catch volumes in alternate locations such as Bahìa Magdalena and Guerrero Negro on the Pacific coast attract local fishers and traders specialized in other species in addition to the squid traders and fishers from Santa Rosalìa and Guaymas (Schneller et al. 2015; Frawley et al. 2019c). Fishery permits are required to land and commercialize catches in Mexico, squid included (Basurto et al. 2013). They are bound to municipalities and often owned by traders (Basurto et al. 2013). Consequently, fishers without permits have to sell to traders to access the market and processing plants.

The squid fishery is a crucial source of income and regional economic development (SAGARPA 2017). However, the benefits from squid catches are distributed in a highly unequal way, which has been identified as a key challenge in the fishery (Cruz-González et al. 2011; Zavala et al. 2005). During peak landings, the squid fishery was ranked fourth by volume in Mexico and was the target of numerous development programs (SAGARPA 2017; Moncaleano-Rubio 2015). Prices that fishers receive are markedly below export price levels, despite limited processing costs. On average, fishers receive 14% of the export prices in Santa Rosalìa, 15% in Guaymas, and 23% in the remaining fishery reporting offices (SI Fig. S2). The fishers’ prices have stagnated or even decreased in some areas. In Guaymas, real prices decreased by 54% between 1995 and 2004 (Cruz-González et al. 2011). The vast majority of fishers consider the prices paid for squid as fixed by traders (82% Guaymas, 94% Santa Rosalìa (Cruz-González et al. 2011)). Intermediary traders, who supply the domestic market, for example, capture the greatest margin of the final product prices (80.8% (Luna Raya 2008)). Experts reported in interviews that they had observed price-setting agreements between squid traders before the beginning of a fishing season. For instance, “… the plants and traders have a talk together and decide we will pay 5 MXN, and then everybody knows it will be 5 MXN ...” (SI Table S1). Traders’ bargaining power is high because they are well-organized amongst each other. The long-term cooperation between traders in the squid fishery, which enables them to fix the prices at which they buy fish below market value, is central in this paper. The cooperation between (mainly Asian) processors and traders has been referred to as an oligopoly (Cruz-González et al. 2011).

Fishers’ prices are affected by their ability to bargain, development programs from the fishery authorities, and climate change. Fishers are less able to organize, in part, due to the ephemeral character of the fishery and their dependence on fishing permits that traders provide (Frawley et al. 2019a). Several official programs address the need for “inclusive development,” for example, development programs implemented under the Humboldt Squid Management Plan (Zavala et al. 2005). One primary development program is about increasing Mexican demand for squid. It recognizes the oligopolistic market structure as a problem and seeks to develop a domestic market for squid products to raise squid fishers’ income (Zavala et al. 2005; Cruz-González et al. 2011).

**Fig. 2:**
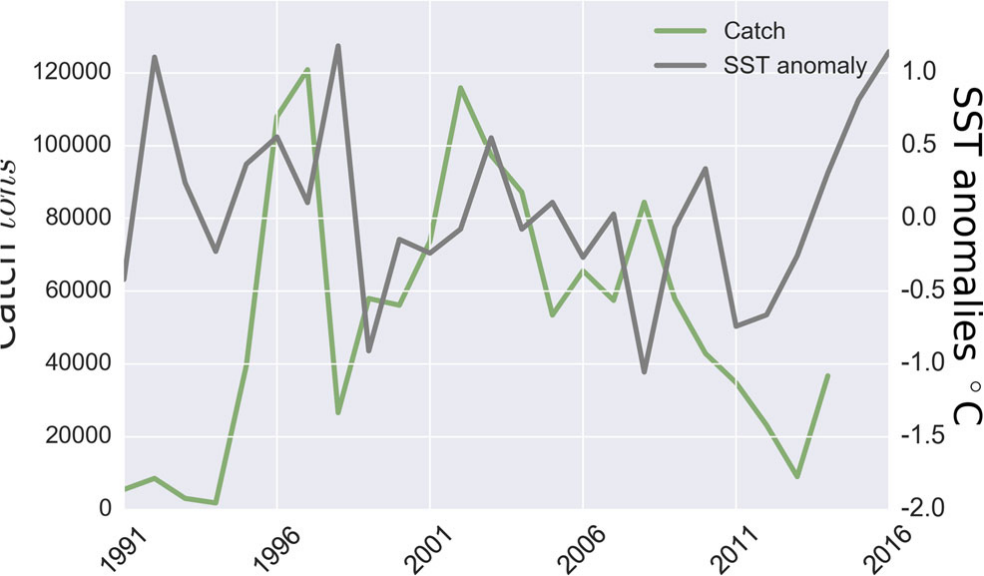
The time series of SST anomalies and catch volume. Low levels of SST are associated with La Niña and high levels with El Niño events

Climate change triggers changes in fishers’ prices, catch volumes, and landing locations (Fig. 1, SI Fig. S3). During years with negative SST anomalies and high primary productivity, high catch volumes and low fishers’ prices typically prevail. Humboldt Squid is then primarily caught in Guaymas and Santa Rosalìa (Fig. 1, 2008). In the transition from negative to positive SST anomalies, catch volumes decline, and fishers’ prices are often higher in Santa Rosalìa and Guaymas but the highest in the remaining fishery reporting offices, where a large proportion of total catches may occur (Fig. 1, 2012). During years with positive SST anomalies, fishers’ prices are intermediate to high, but catch volumes are low, and the fishing fleet largely inactive (Fig. 2, 2015). Overall, the observed mean of SST anomalies increased over the investigated period, while catch volumes subsequently decreased and collapsed by 2015 (SI Fig. S3).

## Three models of the Mexican Humboldt squid fishery

**Fig. 3:**
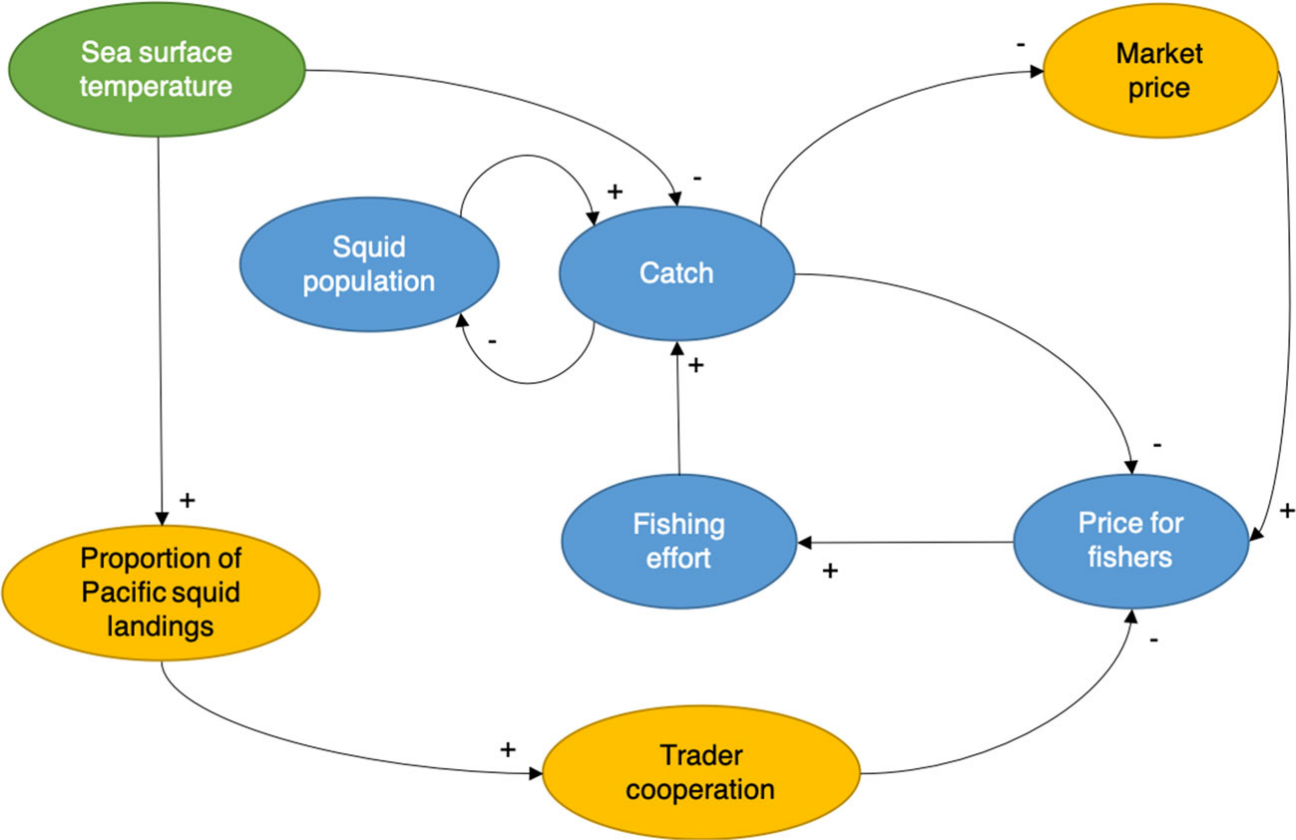
Causal loop diagram and conceptual representation of the three models of the Mexican Humboldt squid fishery in this paper. The models build on one another, adding complexity in each step. They represent social, ecological, and market dynamics. The bioeconomic model (BEM) represents species population, catch, effort, price, and their interactions (blue). The environmental driver model (EDM) adds changes in SST anomalies on catches (green). The social-ecological model (SEM) also includes changes in SST anomalies on the proportion of Pacific squid landings, trader cooperation, and differentiates between fishers’ prices and market prices (orange)

Our analysis aims to investigate small-scale fishery users’ relationships in response to climate change. We focus in particular on the impacts of climate-driven adaptation of target species on trade relationships. For this purpose, we developed a suite of three dynamic models of the squid fishery. To arrive at generalizable insights, we combined previous knowledge from other case studies, theory, and contextualize within the Humboldt squid fishery (Magliocca et al. 2018). We derived our model assumptions, illustrated as a causal loop diagram in Fig. 3, from a combination of literature review, co-authors’ expert knowledge, and in-depth expert interviews. We identified experts through publications, analysis of policy narratives, and recommendations from interviewees, i.e., snowball sampling (Goodman 1961). The squid fishery experts represented a diversity of knowledge fields ranging from commercialization and trade, fishing fleet and practice, climate change, and Humboldt squid (*n* = 8). We followed a semi-structured protocol but allowed for flexible responses through semi-directed and open-ended questions (Huntington 1998).

We then synthesized the causal loop diagram into three systems of difference equations using variables, functional forms, and relationships from theory and empirical data. The data sets included socio-economic, oceanographic, climatic, and fishing data (SI Table S3). We separated the data for conceptualization and parameterization from those for validation (SI Table S3). The resulting system of difference equations represents hypothetical dynamics of all variables, and in particular, their relationships identified in the causal loop diagram (3). Key variables include observed catch volume, location, and fishers’ prices. We provide all functional forms, parameter values, and detailed justifications in the supplementary information (SI S1 and Table S4).

Our baseline model is a bioeconomic model (BEM) of a type often used in real strategic fisheries management (Nielsen et al. 2018). We adopted a functional form from previously published models for each of the bioeconomic variables (Gordon 1954; Burgess et al. 2017; Mansal et al. 2014). From these models, we can provide insights into harvesting strategies, the squid population, and market dynamics. Climate change is frequently represented in fishery management models (Nielsen et al. 2018; Quaas et al. 2016). Our environmental driver model (EDM) incorporates climate change impacts (proxied by SST anomalies) on the squid population to the BEM. This paper’s novelty is the social- ecological model (SEM), which includes social relationship dynamics in response to changes in the fishery, particularly changes in trader cooperation driven by squid response to climate change. For each of the EDM and SEM variables, we derived a discrete functional form building on a mechanistic, empirical, or theoretical understanding of their underlying processes. For instance, we assumed and implemented an exponential break down of trader cooperation following competitors’ new entry from insights based on the theory of cooperation (Casari and Tagliapietra 2018). We coded the models in Python and performed simulations to test development programs’ effects on equitable outcomes. We simulated two development program scenarios, representing current on-going policies identified using narrative policy analysis (Roe 1994) and described in “Do development programs support equitable benefit distribution in the Humboldt Squid fishery?” section.

### BEM—Bioeconomic model

The BEM is based on a discrete Gordon-Schaefer model with a constant growth rate and constant carrying capacity (Gordon 1954). We adapted and calibrated these parameters to match the empirical evidence of the Humboldt squid fishery. We model effort as a function of profit, where increasing profitability leads to higher effort (SI S1 Eq. 2). The market prices are calculated as an isoelastic function, implying that prices respond instantly to changes in traded volumes (SI S1 Eq. 3). We assume the market to be in competitive equilibrium, which means that the fishers’ prices are equal to the market prices after subtraction of processing costs (SI S1 Eq. 4). We calculate income for squid fishers and traders only based on the squid fishery’s income, while we acknowledge income from different sources (Frawley et al. 2019a).

### EDM—Environmental driver model

The EDM builds on the BEM and incorporates the effect of changes in SST anomalies on catches. We use SST anomalies with a trend to represent climate change and a periodic fit to represent climatic variability associated with El Niño and La Niña cycles (SI S1 Eq. 4, Fig. S4). Climate change affects mesoscale water circulation, upwelling, and primary productivity in the Gulf of California (Lluch-Cota et al. 2010; Robinson et al. 2016). SST anomalies can indicate these impacts of climate change. We model the effect of SST anomalies on squid catches as changes in catchability. We use two alternative setups to simulate the model. Either we assume that catchability decreases linearly with decreasing size of individual squid (SI S1 Eq. 6) or increasing SST anomalies (SI S1 Eq. 5). These variables are related, but the squid’s size is a better predictor of catch (SI Fig. S3). First, we assume changes in catchability due to changes in squid size measured in mantle length. Temperate squid phenotypes are long-lived (12–24 months), have large mantle length (60–120 cm), make extended horizontal migrations, and are caught in large volumes. A smaller size (20–40 cm), short-lived (6 months) tropical phenotype has been associated with the limited primary productivity and warm water intrusion characteristic of El-Niño events (Robinson et al. 2016; Hoving et al. 2013; Frawley et al. 2019b). Jig-based fishing methods are less efficient to target this tropical phenotype. Second, based on measurements from acoustic data, we assume changes in catchability rather than in biomass; specifically, acoustic measurements indicate that squid biomass did not decrease proportionally to catch volumes in 2011 (Hoving et al. 2013) or 2013 (unpublished data with K. Benoit-Bird, MBARI) suggesting changes in catchability rather than squid abundance. At present, changes in catch volumes are explained by the described phenotypic changes (Hoving et al. 2013). Further discussion of these interactions is provided in SI S1 and S3.

### SEM—Social-ecological model

The SEM includes the effects of changes in SST anomalies on the proportion of Pacific squid landings, trader cooperation, and fishers’ price, in addition to the variables and interactions in the BEM and EDM. The proportion of Pacific squid landings indicates whether most squid is caught in alternate fishing grounds or the Gulf of California’s core fishing grounds. Based on empirical observations, we assume that the proportion of Pacific landed squid has an inverse exponential relationship to SST anomalies (SI S1 Eq. 7). Temperate squid often migrates seasonally across the Guaymas basin (Hoving et al. 2013). However, migration associated with landings in locations outside the Guaymas basin and the Gulf of California is less frequent and less understood. These landings have correlated with climatic variation and low primary productivity (Robinson et al. 2016). Still, it remains unclear whether these landed squid are individuals from the population found in the Gulf of California or elsewhere.

Fishers’ prices increase if trader cooperation decreases. We assume fishers’ prices to be proportional to market prices and the level of trader cooperation while limited by an empirically estimated maximum and minimum price (SI S1 Eq. 10). Data from Santa Rosalìa and Guaymas suggest that high trader cooperation is associated with low fishers’ prices (Cruz-González et al. 2011; Mascarenas 2017).

Empirical studies have shown that fishers’ prices increase when squid aggregations are found and caught outside of Santa Rosalìa and Guaymas (SI Fig. S2). Hence, we assume trader cooperation to be inversely exponentially related to the proportion of Pacific landed squid. Traders enter regions where two mechanisms reduce their bargaining power: first, not all traders can buy and sell catches in alternate locations because of the geographical limitation of fishing permits (Basurto et al. 2013). Second, squid traders must negotiate the fishers’ prices with the local traders and fishing cooperatives because traders in the alternate locations begin to buy and sell squid (Schneller et al. 2015). In 2010, for example, “... the first wave of new fishers to arrive in Puerto San Carlos had to sell to the permanent canneries, and the sardine canneries even sent their own boats to fish squid with hand lines, the big sardine boats could fit like 20 people.” (SI Table S1). Our function thus represents a deterioration in cooperation through an increase in group size (Casari and Tagliapietra 2018). The group size increases through the inflow of new traders.

## Results

### How well do the models reproduce the catch and price dynamics of the Mexican Humboldt squid fishery?

**Fig. 4:**
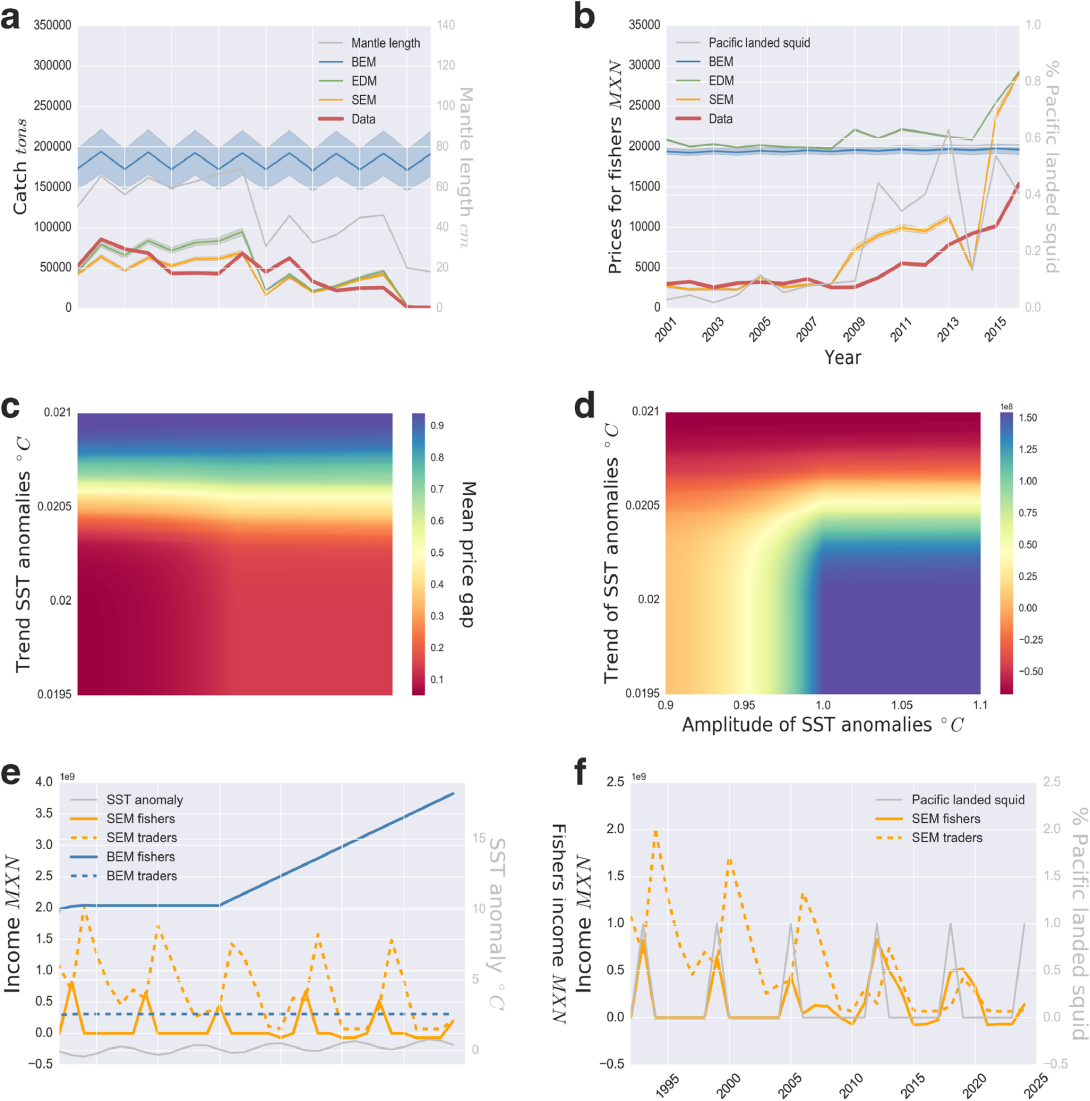
Predicted and measured catch volumes **a** and fishers’ prices **b** for 2001–2016. Predictions of the SEM (yellow), EDM (green), BEM (blue), and measured observations (red). Parameter values and functions are outlined in SI S1, SI Table S2 and S4. The simulations use time series inputs of mantle length (as a proxy for SST anomalies) and proportion of Pacific squid landings (grey, right hand axis). The data represents observations aggregated per year. The models were calibrated via a Monte Carlo process over the range of possible parameter values (SI Table S2). Thick curves represent the mean, and the shaded bands represent the 95% confidence intervals. **c**–**e** The effect of trend and amplitude of SST anomalies on the mean price gap and fishers’ income for SEM simulations. **c** The mean price gap calculated as the ratio between fishers’ prices and traders’ prices (i.e., market prices). The areas in red represent large price differences. **d** Mean fishers’ income. Areas in blue denote high fishers’ income. **e**, **f** Fishers and traders’ income 1990–2025 in two alternate fishery development programs with investments starting in 2005: demand development (E) and ) cooperation development (E). Simulations (**e**, **f**) use the simulated proportion of Pacific squid landings and SST anomalies (grey, right hand axis). **e** A program to increase domestic demand with the SEM (yellow) and BEM (blue). **f** A limitation of trader cooperation (SI S1 Eq. 8) using SEM (yellow)

The SEM yields the best quantitative fit to independent catch and fishers’ prices data from 2001 to 2016 in the Humboldt squid fishery (Fig. 4a and b). To test the models’ performance against observed data, we perform Monte Carlo simulations of the BEM, EDM, and SEM models within the possible parameter ranges. We find that SEM performs best in describing the variance observed in catches and prices (fishers’ prices SEM *r*^2^ = 75.6%; catch SEM *r*^2^ = 60.4%), although these numbers differ marginally with those predicted by the EDM (fishers’ prices EDM *r*^2^ = 74.7%; catch EDM *r*^2^ = 59.3%). In contrast, the BEM predicts both variables poorly in the Monte Carlo simulations (fishers’ prices BEM *r*^2^ = 15.2%; catch BEM *r*^2^ = 3.7%). The SEM and EDM capture qualitative dynamics of catches equally well. Both models also capture the general downward trend and several years of peak catches. Indeed, catch simulations are essentially the same in the second half of the simulated period. Despite large observed price differences, catch values are comparatively close with or without integrating trader cooperation. The form of the effort function causes this proximity. We used an optimization approach to test whether results were sensitive to the effort function (SI S2). Under the assumption of optimizing effort to match the data, the BEM could closely represent catch but not price dynamics (SI S2).

The SEM provides better qualitative predictions of the price level and its increasing trend than the EDM, which overestimates the average prices paid to fishers approximately fourfold. This overestimation results from the competitive equilibrium assumption of this model. Unless the market prices are low, the fishers’ prices do not reach the observed level. This result suggests that the Mexican squid fishery market is not in a competitive equilibrium. Also, the increasing price trend starting in 2010 in the EDM is significantly lagged: the predicted prices only begin to increase during the final 3 years. Price flexibility is the only model parameter affecting prices relative to catch volumes. Thus, changes in catch volumes have to be substantial before the market prices, and consequently, fishers’ prices increase. Lag time for the SEM prediction of increasing fishers’ prices is less prevalent because price flexibility and trader cooperation affect price changes. We conclude that trader cooperation and price flexibility jointly affect fishers’ prices and thus drive price increases. However, the SEM predicts fishers’ prices less well after 2014. First, the price drop in 2014 responds to the decline in the proportion of Pacific squid landings as measured in that year, not reflected in the price data. After 2015, predicted prices of the EDM and SEM increase steeply. The validity of price predictions for all models is limited when catch volumes are close to zero. We further investigate changes in SST anomaly and fishery development programs using the SEM due to its higher prediction accuracy.

### How does climate change affect fishers’ income and price inequality?

Climate change reduces inequality in prices between fishers and traders (Fig. 4c and SI Table S2). First, when mean SST anomalies increase, the prices fishers and traders receive converge. Second, a higher amplitude in SST anomalies leads to earlier convergence between the prices. The main reason for this convergence is changing SST anomalies that affect the proportion of Pacific-landed squid. Higher proportions of Pacific squid landings reduce trader cooperation, which, in turn, increases the prices that fishers receive. If low price inequality is a target, increases in mean SST anomalies are thus desirable.

Increased climate change and variability have contrary effects on mean fishers’ income (Fig. 4d). Elevated SST anomalies are associated with low catch volumes (SI Fig. S5) as well as high fishers’ prices. Translating this result into the squid fishery’s current situation, a long-term trend of increasing SST anomalies could lead to a permanent fishing collapse. There are several mechanisms that, if implemented into the model, may reverse the collapse in catches. We have not included such mechanisms (i.e., the development of alternative fishing gears or a shift in the Pacific Decadal Oscillation) as their influence remains uncertain. Judging from the empirically supported SEM, a critical range for fishers’ income in the squid fishery occurs at low mean SST anomalies and high amplitude. In this climatic range, squid landings from the Pacific trigger convergence in fishers’ prices and market prices while catch volumes are still substantial and generate high income for fishers.

### Do development programs support equitable benefit distribution in the Humboldt Squid fishery?

Inaccurate models can provide misleading guidance for development programs. In the squid fishery, increasing domestic demand may exacerbate already severe income inequalities (Fig. 4e). The BEM and SEM illustrate opposite effects in fishers’ and traders’ income following the Humboldt Squid Management Plan’s domestic demand development programs (Zavala et al. 2005; Luna Raya 2008). The SEM predicts that traders’ income increases with demand, while the fishers’ income remains low, only increasing during years when a high proportion of Pacific squid is landed. Traders directly receive the market price, which increases with demand, but high levels of trader cooperation result in low fishers’ prices. Therefore, fishers are unlikely to benefit from the increase in prices that a higher demand would generate because of the high levels of trader cooperation. In contrast, BEM is unable to predict the changes in traders’ income resulting from increasing demand. In the BEM, the results and thus the guidance for the development program are directly opposite from those in the SEM. Hence, the importance of accurate model conceptualizations to provide correct guidance for fishery development programs.

Furthermore, our SEM simulations show that limiting trader cooperation is more effective than increasing demand to reduce income inequality and increase fishers’ income (Fig. 4f). Indeed, the margin traders can take from the market prices is lower, so fishers receive higher prices. Providing fishery permits to other traders and fishers or increasing fishers’ ability to self-organize could be ways to increase fishers’ bargaining power and thereby realize such a program (Zavala et al. 2005). Additionally, if the reduction in squid landings driven by climate occurs earlier than predicted here (further discussion SI S3), limiting trader cooperation in combination with increasing demand might reduce short-term losses in fishers’ income from the lower catch volumes.

## Discussion and conclusions

Social relationship dynamics can stabilize livelihood outcomes in fisheries—ignoring these dynamics in decision support for management could, therefore, exacerbate inequalities in the Mexican Humboldt squid fishery. Understanding the future of fishery livelihoods impacted by climate change requires knowing their effect on fishery users’ individual behavior and their cooperative and competitive relationships. Previous work had found that climate change drives fishers’ income (Cinner et al. 2012). Additionally, our results highlight that the effects of climate change on cooperative trade relationships are essential to determine changes in fishers’ income. Using empirical data and a dynamic SEM, we show that climate change and variability have balancing effects in the Mexican Humboldt squid fishery. If the mean SST anomalies increase, fishers benefit from higher prices due to reduced trader cooperation while catch volumes significantly decrease. Conclusions about the benefits and losses for fishers and traders due to climate change are contingent on the complex trade-off between the effect of climate change at the individual and collective levels.

Development programs that omit relationships between fishery users can have negative consequences. Addressing the impacts of such relationships can help develop carefully crafted policies. Our SEM simulations show that increasing demand would not increase fishers’ income in the Humboldt squid fishery. This result contrasts with what the BEM suggested, based exclusively on supply and demand mechanisms, which determine prices. However, the BEM fails to explain price formation in the Humboldt squid fishery because it ignores traders’ price setting behavior. In other social-ecological systems, models that omit social relationships have misguided managers in the past (Degnbol and McCay 2007). Although many of squid fishery characteristics are idiosyncratic, cooperative and competitive relationships between and amongst fishers and traders and their influence on fishers’ livelihoods are pervasive in small-scale fisheries (Ferrol-Schulte et al. 2014; Drury O’Neill et al. 2018). These challenge the efficiency and efficacy of development programs that omit such relationships in their planning.

Recognizing the effects of relationship dynamics on fishery livelihoods can support carefully crafted fishery development programs. Our SEM simulations of the Humboldt squid fishery illustrate that increasing demand without mitigating the market power impacts of cooperative trade relationships can exacerbate existing income inequalities. Increasing demand does not achieve inclusive development by increasing economic benefits for fishers required by the fisheries management plan (*Humboldt Squid Management Plan* (Zavala et al. 2005)). In contrast, an alternative, simulated program to reduce trader cooperation reduces traders’ bargaining power and has a direct and positive effect on fishers’ income in line with inclusive development. Providing licenses to new traders and fishers to sell and buy squid could reduce the problem. In reality, the powerful traders (and processors) who benefit from the current market configuration are likely to pose major obstacles to providing new licenses (Luna Raya 2008). Therefore, we expect that appropriate timing for intervention is important (Walker et al. 2020). During an El Niño period, we would expect this intervention to be more feasible because there is already a group of new entrant traders and fishers who also buy and sell squid and reduce current traders power in the fishery. However, if this turns out to be politically unfeasible, an alternative could provide special trading rights to the current traders, contingent on carefully negotiated minimum price levels for fishers. An equal benefit distribution in the fishery would improve fishers’ livelihoods and affect fishers’ interaction with the target species. These must be considered beforehand.

We call for explicit inclusion of cooperative relationship dynamics in management models of small-scale fisheries. It is impossible to predict income and income inequality in the squid fishery without explicitly accounting for relationships between fishery users. Our simulations show that accurately predicting fishers’ prices relies on integrating dynamic trade relationships driven by adaptations of the squid population to climate change. The influence of trade relationships on prices and price transmission has been documented frequently in small-scale fisheries (Drury O’Neill et al. 2018; Wamukota et al. 2014; Elsler 2020). While many different mechanisms could explain this influence, our general insight is that social relationship dynamics are necessary to predict price dynamics. In our case study, catch levels were similarly well explained by the simpler EDM. However, we expect that predicting catch and population dynamics will depend on the influence of social relationships on fishing practices in many other small-scale fisheries (Ferrol-Schulte et al. 2014).

Conceptualizing and implementing cooperative and competitive relationships in small-scale fisheries’ predictive models provide a social-ecological management perspective, but this does not come without challenge. We foresee two main challenges for modeling other social-ecological systems. First, in our case, the proportion of landed squid was the main driver of trade relationships. Instead, these relationships depend mostly on internal mechanisms between fishery users, such as individual and reciprocal motivations. It becomes necessary to understand these mechanisms either through empirical studies or by inferring from theory. To this end, game theory and industrial organization have identified candidate mechanisms such as utility-based decisions and group size dynamics (Axelrod et al. 1995; Casari and Tagliapietra 2018). The second challenge is methodological. The iterative and interdisciplinary approach needed to conceptualize genuinely integrated social-ecological models is time consuming. It requires the active participation of experts of parts of the system and navigating their willingness to collaborate.

Social-ecological modeling and its interdisciplinary approaches can be a useful vehicle to bridge the gap between current bioeconomic models and the reality in which social relationship dynamics shape fishery outcomes. Social-ecological models, like the one presented here, blend bioeconomic approaches with ecological models and knowledge from social science (Schlüter et al. 2012). The development and adoption of these tools are rapidly growing (Schlüter et al. 2012). These models enable us to challenge the predictive capacity of models that omit the role of social relationship dynamics in response to climate change. Prediction accuracy is one important element for models to be useful in guiding for formal fisheries management. Our analysis provides the first, critical step towards the inclusion of social relationship dynamics by advancing social-ecological systems models in a formal fisheries management context. Future work could further develop this approach by incorporating relevant data on fishery users’ relationships and systematically adapt the analysis to various fishery contexts to support development programs sensitive to informal cooperative and competitive dynamics.

## Supplementary Information

ESM 1 (PDF 640 KB)
