# Supplementary material for: Social relationship dynamics mediate climate impacts on income inequality: evidence from the Mexican Humboldt squid fishery
Source: Reg Environ Change. 2021 Mar 24;21(2):35. doi: 10.1007/s10113-021-01747-5 (PMC8550063; doi:10.1007/s10113-021-01747-5)
Supplement: Supplementary file 1 — (PDF 640 KB) [file 10113_2021_1747_MOESM1_ESM.pdf]

Supplementary Information for

Social relationship dynamics mediate climatic impacts on income inequality: evidence from the Humboldt squid fishery

This PDF file includes:

Supplementary text
Figs. S1 to S5
Tables S1 to S4
References for SI reference citations

Supporting Information Text

S1

Models Our models are based on a bioeconomic approach (1, 2), where several variables such as target species (squid) population, effort and price describe system dynamics. We present three models, ordered by increasing levels of complexity.

Bioeconomic Model (BEM) represents fishery dynamics without environmental drivers and multilevel interactions. This is the simplest of our models and it tracks changes in the squid population, effort and prices.

Population dynamics. Net recruitment and harvest of Humboldt Squid was modeled using a discrete yearly Gordon-Schaefer model, following (3):

$$S_{t+1} = S_t + gS_t\left(1 - \frac{S_t}{K}\right) - C_t, \quad [1]$$

where the density-dependent growth of the squid population S_t is determined by an intrinsic growth rate g and carrying capacity K, which we assume to be constant. The harvest based mortality C_t is given by the catch $C_t = qE_tS_t$, where q denotes catchability and E_t denotes fishing effort at time t. The yearly intervals imply a single annual reproductive cycle, corresponding to the average age-at-maturity of phenotypes, see (4).

Fishing effort. We assume that fishing effort increases at a rate proportional to the difference between revenue and cost (2):

$$E_{t+1} = E_t + \mu\left(qP_{f,t}S_tE_t - \sigma E_t\right), \quad [2]$$

where P_f is the fisher' price and σ is the cost per unit effort. In order to have positive fishing effort for all levels of prices and squid numbers, we assume that $\sigma < 1$. The rate of change of effort is of unit MXN, thus can be interpreted in such a way that the fishery is profitable when $dE/dt > 0$ and losing money when $dE/dt < 0$. μ therefore captures effort adjustment to profit changes and has the unit $1/MXN$. We define $\mu \equiv 1$ to normalize $E \in [0, 1]$.

Prices. We follow (5) in the representation of the price curve. The Humboldt squid is primarily an export product (6) therefore we fit the curve to export price data. The fishers' price is then provided by:

$$P_{f,t} = \gamma C_t^{-\beta} - \kappa, \quad C_t \geq 0, \quad [3]$$

where the first part of the equation provides the market price including C_t is the catch, β represents constant price flexibility and the scaling coefficient γ determines demand. The fishers' price is then given by a slight variation κ which represents the cost of transport and processing. Following classical economic theory one price prevails in a market and is differentiated only by processing and transport cost (7).

Environmental Driver Model (EDM) is obtained from BEM by including climatic changes and variation into bioeconomic model.

Sea surface temperature anomalies. We first set up climatic changes and variation as a periodic function fitted to sea surface temperature (SST) anomalies (*figure S4*):

$$T_t = a_0 + a_1t + a_4(a_2\cos t + a_3\sin t), \quad [4]$$

where T is the observed SST anomalies in the GOC and the parameters a_i, $i = 0, 1, 2, 3$ represent the best fit to mean, amplitude and period of interannual cycles. a_4 is used in the simulations to modify amplitude of the function (*table S2*).

Squid responses. Squid responses to temperature changes are reflected in changes of phenotype and migration. There are two phenotypes that develop dependent on the temperature they encounter in juvenile state (4). Smaller, tropical squid that develops during warm water conditions has reduced catchability in comparison to large, temperate squid (*section S3*). This is represented in our model by using variable catchability q_t:

$$q_t = q_c\frac{T_{max} - T_t}{T_{max} - T_{min}}, \quad [5]$$

where q_c is the maximum catchability coefficient, T_{max} the maximal expected temperature and T_{min} is the minimal expected temperature. The second term allows catchability q_t to vary between 0 and q_c when temperature varies between T_{max} and T_{min}. For simulations *figure 3 A, B* (main text) we use mantle length (ML) data timeseries to calculate q_t, the reasons for this are further discussed in *section* S3:

$$q_t = l - kL_t, \quad [6]$$

where k and l are the coefficients relating oceanographic variation via the implied mantle length L relationship to catchability (*figure S3b*). To guarantee positivity of q_t, the coefficients k and l has to satisfy $l > kL_{\max}$.

Social-ecological Model (SEM) includes the second response of squids to temperature dynamics is the migration of temperate phenotypes when they encounter unproductive, warm water (8). We capture the fishery relevant changes in migration through landings from inside and outside the Gulf of California. We fit a function driven by SST anomaly to mimic peaks of landings outside the GOC (figure S4):

$$M_t = \alpha e^{\lambda(a_2\cos t + a_3\sin t)}, \quad [7]$$

where the proportion of captured migrated squid M_t is determined by the SST anomalies T_t in a given year. The parameter α is used to scale $M \in [0, 1]$ and λ regulates the incline of migration peaks.

Trader cooperation. Trader cooperation enables traders to set prices for fishers in the usual fishing locations (9). The level of trader cooperation is contingent on the ability of traders to organize. They are able to organize well in usual fishing locations but are less able when migrating to new fishing locations with existing fishing and trade structures (10). Therefore we assume trader cooperation to be driven by the proportion of migrated squid. We choose an exponential form to represent fast collapse of cooperation beyond a tipping point observed for cooperation in groups (11). Thus trader cooperation is an inverse, exponential relationship to the proportion of migrated squid M_t:

$$R_t = e^{-\delta M_t} \tag{8}$$

$R_t \in (0, 1]$, where 0 means no cooperation, 1 means full cooperation and δ scales between traders that operate in the squid fishery in a given year.

Price. The fishers' price is affected by the level of cooperation between traders. For this model it is useful to differentiate between market price P_m and fishers' price P_f. The market price P_m as follows:

$$P_{m,t} = \gamma C_t^{-\beta}, \quad C_t \geq 0, \tag{9}$$

where the parameters are as introduced for equation 3. The theoretical maximum price the fishers receive is the market price reduced by transport and processing costs, see (12) and equation (3). The theoretical minimum price the fishers receive, before they exit the fishery, corresponds to the cost of fishing (13). We assume the prices fishers receive are dependent on trader cooperation. Expression for $P_{f,t}$ is obtained by modifying equation (3) and reads as:

$$P_{f,t} = (1 - R_t)(P_{m,t} - \kappa) + R_t \frac{E_t \sigma}{C_t}, \tag{10}$$

where the first term determines the proportion of catches sold at theoretical market price, in locations outside the historical centers of the squid fishery. The second term determines the proportion R_t of catches sold at production cost $\frac{E\sigma}{C}$ in Santa Rosalìa and Guaymas.

Revenue and income. The income of fishers is a function of fishers gross revenue and fishing expenses:

$$I_{f,t} = C_t P_{f,t} - \sigma E_t \tag{11}$$

Traders income is a function of gross revenue (i.e. biomass sold at market prices) reduced by prices paid to fishers and processing costs of biomass:

$$I_{t,t} = C_t P_{m,t} - C_t P_{f,t} - \kappa C_t. \tag{12}$$

S2

Optimization approach. The poor fit of the BEM model to both the catch and fishers' price (figure 3i, main text) can be explained as a consequence of two separate properties of the model. First, fishers' price is represented via a simple exponential of the form $\gamma C^{-\beta}$ of which we fix γ and β (*maximum demand* and *slope of price flexibility*, respectively) to empirically derived values. C, not being a free parameter the remaining coefficients cannot represent the strong response of the price data observed in the fishery. Second, the catch correspondence in the BEM model is described by the relation between squid population S and effort E and the constant factor q. Effort being constrained by the fishers' price P_f in combination with squid population S and q itself, creates a high dependency on the effort function. To further investigate effort and support the Monte Carlo analysis we perform a numerical, non-linear optimization of the three models where effort is a normalised, free floating variable of the system.

We build a Julia (14) program which used the Ipopt (15) non-linear solver as a part of the JuMP (16) mathematical optimization package. All values which could be identified empirically were configured as fixed values in the optimization, and those that could be bounded by empirical data had said bounds applied in accordance with *Table S2*. The non-linear objective function is a sum of least squares match to the empirical catch C and price for fishers P_f found in *figure S3*, which leaves the following parameters and bounds for the optimization of the BEM model:

$$
\begin{aligned}
0.0 \leq\, & g \leq 3.2 && \text{Squid population growth rate}\\
1000.0 \leq\, & \kappa \leq 2148.0 && \text{Cost of processing and transport}\\
0.0 \leq\, & E(t) \leq 1.0 && \text{Effort}\\
K-(0.3K) \leq\, & S(t) \leq K+(0.3K) && \text{Squid population}\\
& P_f && \text{Fishers' price}\\
& C(t) \geq 0.0 && \text{Catch}\\
& P_m \geq 0.0 && \text{Market price.}
\end{aligned}
$$

Four constraints satisfy the BEM model, the top three being non-linear

$$S(t+1) = S(t)\left[gS(t)\left(1-\frac{S(t)}{K}\right)-C(t)\right] \qquad [13]$$

$$p_m(t) = \gamma_m C(t)^{-\beta} \qquad [14]$$

$$C(t) = qE(t)S(t) \qquad [15]$$

$$p_f(t) = p_m(t) - \kappa \qquad [16]$$

To obtain results for the EDM and SEM models, the time dependent q_t (5) is substituted into (15), and the additional equations described in the EDM and SEM sections above are added. Figure S1 displays the results of the optimisation and can be compared directly to *figure 3i* in the main text.

It is not surprising to see a drastic improvement in the match to the BEM's catch prediction here. As described above, most of the BEM model is constrained via empirical values. Now that effort is no longer fixed, it makes sense for the optimiser to arrange effort to directly correspond to the profile of catch. This improvement in catch is transferred to fishers' price but only achieves minimal gain due to the fixed coefficients discussed above. We can therefore conclude that even an *optimal effort equation* would not enable the BEM model to represent this fishery.

Improvements in the matches of the EDM and SEM to the catch data are visible, although outliers are also introduced (notably 2002 in the SEM model which is a direct consequence of an 'optimal' effort of 0 that year). Fishers' price values are similar, although the 2015–16 prices overshoot as a result of catch estimates close to 0 in this period for both models. These small differences in results allow us to posit that the current analytical effort function compared to an optimal numerical effort function does not need to be considered a 1st order correction, since it does not play as much of a role in achieving an acceptable result as the trader cooperation addition does. Thus the additional overhead that comes with a non-analytic, unknown function as a part of the model is not warranted, which validates the Monte Carlo methodology presented in the main text as the preferred implementation for our models.

S3

Ocean dynamics, squid ecology and squid catches. Here we describe in greater depth the interactions between climate driven oceanographic variables, squid ecology and catch volumes observed in the squid fishery. Then we will continue the discussion of their implementation in the model.

Squids' phenotypic plasticity enables radically different size-at-maturity and growth rate in individuals (17). In various contexts ecologically relevant oceanographic variables such as water temperature at various depths and primary productivity (food availability) driven by coastal upwelling, wind intensity and La Niña cycles have been related to different phenotypic expressions and fishery relevant variables such as squid catch volume and location (4, 8, 18, 19).

During the La Niña conditions which followed the 97-98 El Niño event, reduced SST anomalies and high coastal upwelling led to above average captures (18). Squid phenotypes associated with temperate, productive oceanographic conditions can live 12-24 months, achieve large body size (pre-2009 mean mantle length at maturity 63.0 ± 8.6cm SD, n=101) and are able to make extended horizontal migrations (4, 8, 19).

In contrast, warm water temperatures on the surface and throughout the upper 100 m of the water column were observed during the 2009-10 El Niño event across much of the Gulf of California and within the Guaymas Basin in particular (4). Historically low catch volumes were observed similar to the El Niño event of 1997-1998 (*figure S3d*). During warm water ENSO events squid in the Guaymas Basin live $\tilde{6}$ months, reaching a mantle length at maturity of 20-40 cm, and are found primarily in the open waters rather than coastal shelf areas (4).

Despite large reductions in fisheries landings following El Niño 2009-2010, limited acoustic data suggests that squid biomass did not decrease in proportion to landings in 2011 (4) or 2013 (unpublished data with K. Benoit-Bird, MBARI). This suggests that the relation between phenotypic expression and catch volume is more complex than a simple reduction in biomass. Today small squid size-at-maturity is thought to primarily affect catchability likely conditioned by two factors, first, current jig-based catch methods using 15-20cm luminescent jigs may be inadequate to catch comparatively small, tropical phenotype squids. Second, different phenotypes of Humboldt squid exhibit different patterns and pathways of migration (4, 20). The tropical phenotype appears to disperse from the shelf habitats favored by the temperate phenotypes (4) and the everyday operations of small-scale fishers with 3-5m fiber boats are limited to $\tilde{1}0$ nautical miles.

In the main text we employ two ways to attain catch volumes from the models. First, we calculate SST anomalies, catchability and catch volumes employing above described equations (*equations 4* & 5). Previously, the relationship between SST anomalies and squid size-at-maturity has been established (4). However, the nature of this relationship is complex. Due to the complex nature of multiple oceanographic variables interacting to change squid phenotype reliance on a single variable such as SST anomalies is necessarily limited. The correlation we establish between SST anomalies and squid mantle length is $r = -0.230$. The simulations (main text *Fig. 3i C–F*) thus continue to predict income and catch volumes after 2015 which is in contrast to recent observations ((21).

To improve these predictions we use input of mantle-length timeseries to drive the model (*figure 3i A&B*). For these simulations we employ *equations 4* & 6 as described in *section 1*. These simulations provide a good approximation of catches because catch volume in the Guaymas basin is highly correlated with squid-size-at maturity ($r^2 = 0.738$, (22)).

SST anomalies and squid mantle length. The literature points to the correlation between SST anomalies and squid mantle length. We analyzed the correlation between SST anomalies and squid mantle length at maturity. We interpolated to a monthly timeseries for SST anomalies and mantle length data due to the irregular sampling of mantle length data. A negative lag of 4 months for SST anomalies yields the best correlation ($r = -0.230$), indicating that a change in oceanographic conditions initiates a change in the squid population.

Having established that SST anomalies best correlate with mantle length, we fitted a periodic function to the SST anomalies with a period that approximate La Niña cycles. There is some ambiguity to the precise period as La Niña does not occur with precise regularity. To address this we take an empirical approach by fitting a range of possible periods and choosing the period that best fits the data ($r^2 = 0.8$). We find that a period of approximately 6.5 years best fits the data. The form of the fitted model is provided in equation 4.

S4

Data availability and data preparation.

SST SST anomalies data was accessed through the NOAA CoastWatch (https://coastwatch.pfeg.noaa.gov/) the Pathfinder project (Pathfinder v5, September 1981- December 2009, 0.05°) provides timeseries of the Moderate resolution Imaging Spectroradiometer (MODIS, July 2002-2015, 0.0125 o) and the Advanced Very High Resolution Radiometer (POES AVHRR, April 2007-2015, 0 .0125°) as well as Optimum Interpolation Sea Surface Temperature (September 1981-2017, 0.25°). SST anomalies data was geographically constrained to the Guaymas basin by taking the spatial mean of locations within the Guaymas basin. We averaged values of geographical overlaps, however, these values were found to be similar for the entire GOC and the Guaymas Basin. We removed monthly climatological means to calculate yearly averages from September 1981 to 2015 of the observed values.

Mexico's National fisheries statistics was obtained from dataMares. Data was filtered to only contain values for squid and were in doubt that the species was Humboldt squid, values were removed. Data of catch volume and value was aggregated to years. Value was inflation-adjusted to the year 2017 using Bank of Mexico consumer price index (23). The same procedure was applied to gas price data (24).

UN ComTrade data for export statistics was downloaded from *https://comtrade.un.org/data/*. The data was filtered for Mexico and squid. The low species resolution of the data does not allow us to exclude other squid species than Humboldt squid. However, catch volumes of other squid species are by several magnitudes smaller to that of Humboldt squid and therefore would only have a limited effect on our calculations. We converted the currency to MXN pesos using the exchange rates of the 1st January of any given year provided by *https://www.oanda.com/currency/converter/*. Values were then inflation-adjusted to the year 2017 using Bank of Mexico consumer price index (23). We aggregated to yearly values of catch volumes and averaged prices weighted by volume. We use R to determine the nonlinear (weighted) least-squares estimates of the parameters of the export price function 9. The fitting achieves parameters as reported in *table S2* and a residual sum-of-squares: 5.72e08.

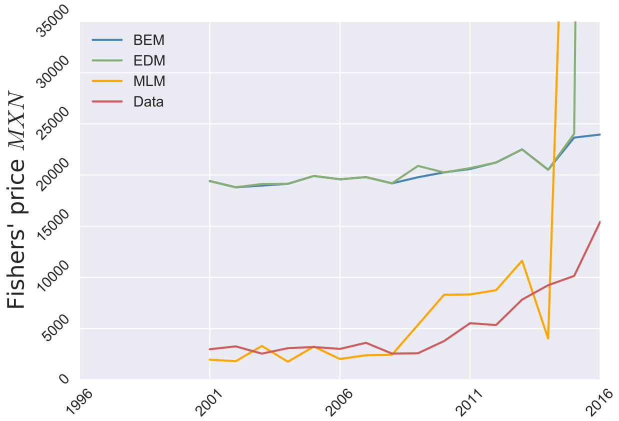

(a) Fishers' price P_f.

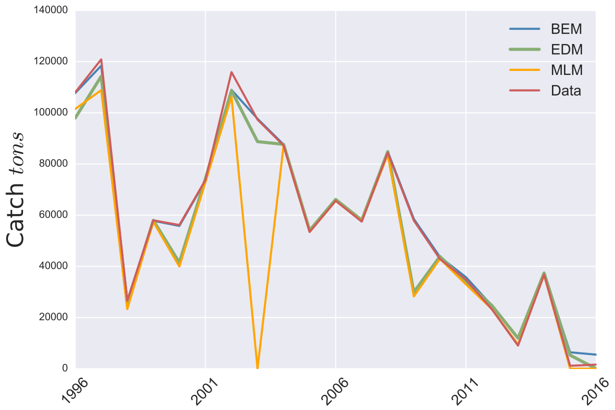

(b) Catch C.

Fig. S1. Predicted and observed catch C and fishers' price P_f are displayed between 1996-2016 using a non-linear optimisation with free floating effort. Compared to Figure 3(i) (main text) the catch is better represented across all models in most places: significantly in the BEM, but only slightly in EDM and SEM. Fishers' price remains qualitatively similar across the models with only a slight improvement in the BEM.

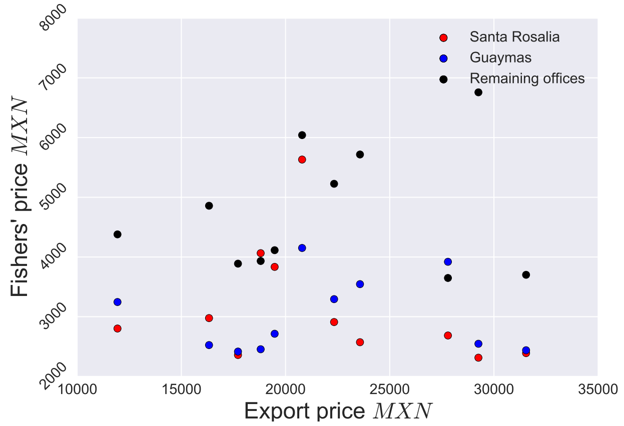

(a) export price data P_m and fishers' price P_f as aggregate values per year. Data source UN ComTrade and Mexican fishery statistics see Table S3.

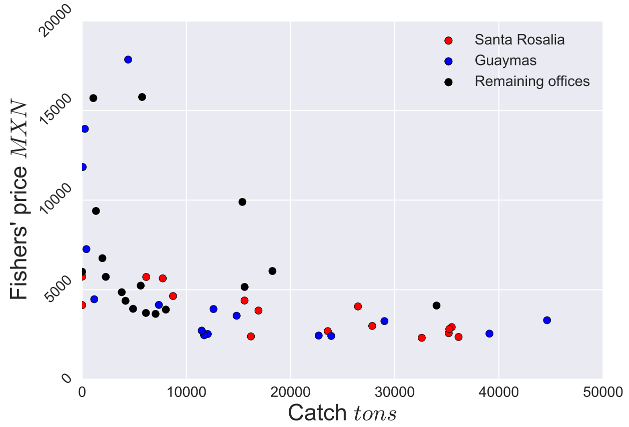

(b) prices for fishers and volumes sold in Guaymas, Santa Rosalìa and remaining fishery reporting offices. Data source (25) see also Table S3.

Fig. S2. The distribution of revenues in the Mexican Humboldt squid fishery is contingent on the prices fishers receive compared to the domestic or export market price. The prices differ distinctly by fishery reporting office. We distinguish between Santa Rosalìa, Guaymas and the remaining fishery reporting offices. Fishers in Santa Rosalìa receive on average 14% , in Guaymas 15%, and in the in remaining fishery reporting offices 23% of the export price. Similarly, prices per ton are lowest in Santa Rosalìa reporting office.

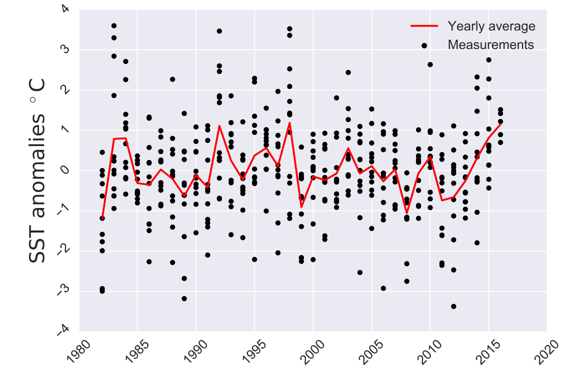

(a) timeseries of individual measurements and yearly average sea surface temperature (SST) anomalies per year from 1981–2016

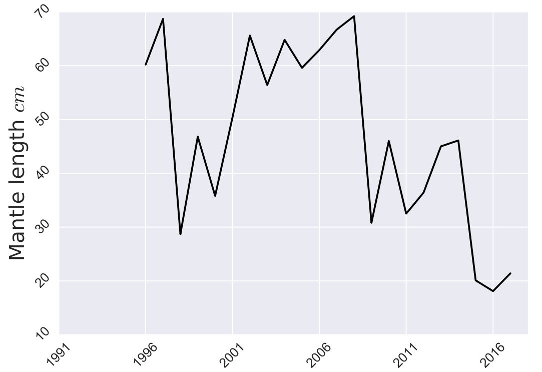

(b) timeseries of average mantle length of squid at maturity per year from 1996-2017

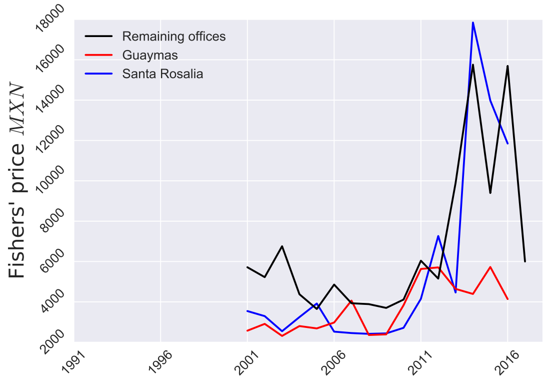

(c) timeseries of per year aggregate fishers price P_f from 2001-2016

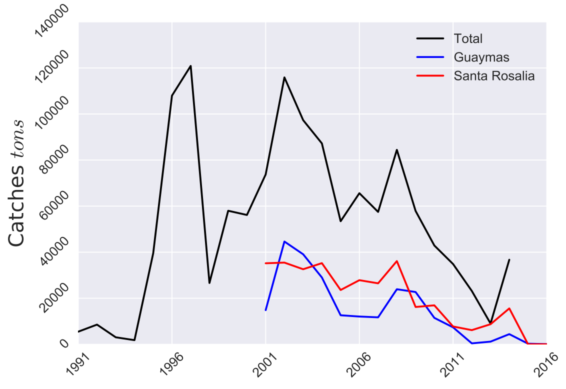

(d) timeseries of per year aggregate catch C from 2001-2016

Fig. S3. timeseries of per year aggregate value of social-ecological dynamics in the squid fishery, representing SST anomaly, fishers' price and catch volumes in different locations. Oceanographic variation leads to changes in fishers' price, catch volumes and landing location. Sources of data sets see Table S3.

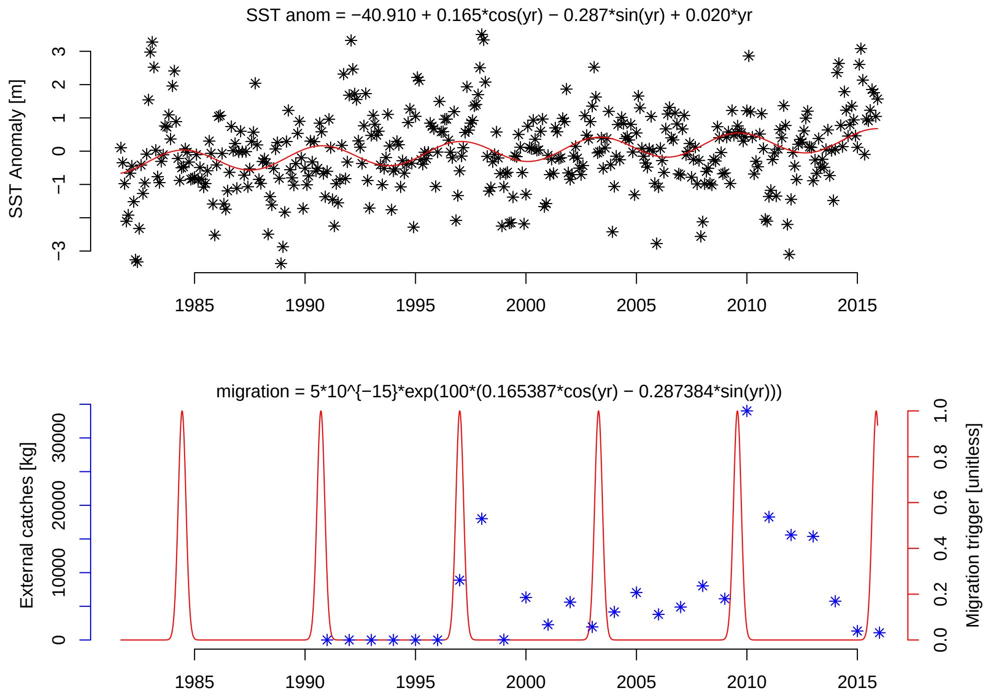

Fig. S4. Upper plot presents chosen periodic fit of the temperature function to sea surface temperature (SST) anomalies from 1981-2016. Lower plot derives a spiked migration function that is driven by sea surface temperature anomaly peaks compared to proportion of landings outside the Gulf from 1990-2016.

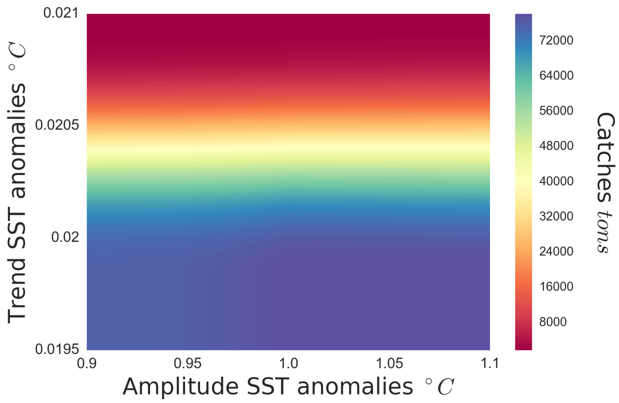

Fig. S5. complementary figure to simulations of changes in SST anomaly trend and amplitude in the main text - displaying mean catch volume for simulations over the parameter ranges representing SST anomaly trend a_1 and variability a_4 (Equation 4).

Tables

Table S1. Table of expert interview respondents in semi-structured interviews.

	Interviewee type of stakeholder	Experience
1	Marine ecologist (PhD), CIBNOR	Ecology of squid under oceanographic change
2	Fisher, permit holder and active in Comitè Sistema de Producto del Calamar Sonora	Several decades of fishing amd commercialization experience, policy negotiations
3	Natural resource researcher for policy support (PhD), INAPESCA	Socio-economic analysis of the squid fishery
4	U.S. sustainability researcher and lecturer (Professor)	Fishing of squid aggregation
5	Economist (Professor), UABCS	Economic fishing activities in the Gulf, rentability of squid fishery
6	Marine resource researcher (MSc), CICIMAR	Squid market and value chain analysis
7	Marine scientist (Professor), UABC	Oceanographic changes in the Gulf of California
8	Fisheries policy consultant (PhD), UABC/ Instituto Nacional de Pesca	Fishing dynamics and viability of squid fishery in Pacific

Table S2. Parameter ranges and values

Parameter	Description	Standard value used	Unit	Source/Reference	Method	Modified value for simulations
g	squid population growth rate	1.4	$tons/year$	(26)	mean value from publication	0–3.2 in optimization and *figure 3 A,B* main text
K	carrying capacity	1208770	$tons$	(25)	estimated from maximum catches	K $\pm$ 30% in optimization
σ	cost per unit effort all fleet	107291548	MXN/maximum fleet effort *	Gas prices (24), boat trips per season (6), fuel consumption per trip (27)	-	50907027–212300758 in *figure 3 A,B* main text
q	catchability	q_c = 0.1	-	assumed	-	constant q_c changes to variable q_t in EDM, SEM
$gamma$	maximum demand	49200	$tons$	UN ComTrade	fitting	2000–51000, simulation *figure 3 E* main text
$beta$	slope of export price function	0.0736	MXN/$tons$ *	UN ComTrade	fitting	-
κ	cost of processing and transport	1776.25	MXN/ton	(6)	-	1000–2148 in *figure 3 A,B* main text (estimated from minimum export price, UN ComTrade)
a_0	SST anomaly y-intersect	-40.9079	$^\circ C$	figure S4	fitting	-
a_1	SST anomaly trend	0.020464	$^\circ C$	figure S4	fitting	0.0195–0.021 in *figure 3 C,D* main text
a_2	SST anomaly variability	0.165387	$^\circ C$	figure S4	fitting	-
a_3	SST anomaly variability	-0.287384	$^\circ C$	figure S4	fitting	-
a_4	SST anomaly variability	1	$^\circ C$	figure S4	fitting	0.5-1.5 in *figure 3 C,D* main text
T_{max}	scaling catchability	0.5262	$^\circ C$	-	calculated from SST anomalies simulations 1990-2015	-
T_{min}	scaling catchability	-0.4311	$^\circ C$	-	calculated from SST anomalies simulations 1990-2015	-
l	catchability intersect	-0.0318	-	Mantle length dataset	-	calculated using mantle length input
k	catchability slope	0.0018	-	Mantle length dataset	-	calculated using mantle length input
α	proportion of migrated squid	3.9e-15	-	-	calculated against reference T_{max}	-
δ	scaling trader cooperation	1	-	-	assumed	-

*all prices used are in MXN inflation adjusted to 2017

Table S3. Data and use in model

Data	Associated variables	Spatial resolution	Temporal resolution	Time span	Use
Trip tickets	catch C, effort E, price P_f	Baja California Sur	per trip	1997–2014	Cross-validation C, P_f, validation E
Mexico's National fisheries statistics, 2001-2016	catch C, migration M, price P_f	Mexico	monthly	2001–2016	Parameterization M, validation C, P_f, model simulation input M
Expert interviews	trader cooperation R, price P_f, catchability q	North-West Mexico	-	-	Model implementation
UN ComTrade	price P_m	World	per trade	1991–2014	Parameterization of P_m
Annuarios pesqueros	price P_m, catch C	Mexico	annual	1997–2013	Cross-validation UN ComTrade, Mexico´s National fisheries statistics
NOAA Coastwatch	SST anomaly T	Guaymas basin	multiple per year	1981–2016	Model implementation and parameterization T
Mantle length measurements	catchability q	Guaymas basin	multiple per year	1996–2017	model simulation input q
Mexican gas prices	cost per unit effort σ	Mexico	annual	1996–2016	Parameter estimation σ

Table S4. Assumptions of functional forms.

Equation	Description	Assumptions	Source/Reference	Explanation of model and parameter choice
Equation 1	logistic function of squid population dynamics	the biomass of the squid population dynamics can be represented by a logistic growth function where the parameters are held constant	(3, 28)	the growth of the population is determined by an intrinsic growth rate g and cannot grow beyond the carrying capacity K. The squid population is linearly affected by loss through harvest
Equation 2	fishing effort	fishing effort increases at a rate proportional to the difference between revenue and cost	(2)	follows the heuristic of bounded rationality in system dynamics models corresponding with empirical evidence from fisheries
Equation 9	isoelastic market price	price flexibility beta is fixed over time and price changes directly with catch levels C, demand is given by the scaling coefficient γ	Model of price flexibility is considered a more appropriate empirical demand measure for harvests than demand elasticity (29)	decreasing price over volume
Equation 3	fishers' price	we assume the law of one price prevails in the market and prices only by processing and transport cost	(7)	P_f is linearly dependent on export market price P_m reduced by the cost factor κ
Equation 4	periodic function with a trend of temperature change proxied by SST anomaly	SST anomaly represents changes of temperature and productivity in the environment associated with cyclical La Niña/El Niño fluctuations		we fit a periodic function to existing data points on SST anomaly
Equation 6	normalized function of catchability	catchability can explain the difference in catch volumes between phenotypes. The catchability of the squid population is linearly related to oceanographic variation represented in sea surface temperature. We assume high catchability of large, temperate phenotypes explaining high catch volumes which develop during below average sea surface temperature.	empirical observation, (8)	squids adapt their phenotype to ambient temperature and productivity. In warm, unproductive conditions a tropical phenotype develops where we observe low catches using current fishing techniques and high catches during cold, productive conditions
Equation 7	linear function of catchability	catchability can explain the difference in catch volumes between phenotypes. The catchability of the squid population is linearly related to changes in mantle length, we assume high catchability of large, temperate phenotypes explaining high catch volumes	empirical observation, (4, 8)	squids adapt their phenotype to ambient temperature and productivity. In warm, unproductive conditions a tropical phenotype develops where we observe low catches using current fishing techniques and high catches during cold, productive conditions
Equation 8	proportion of captured migrated squid	determined by change in sea surface temperature captured in SST anomaly T, $M \in [0, 1]$	model fitted to data of catch location outside the Gulf of California (figure S4)	the migration of squid aggregations associated with landings from locations outside the Guaymas basin is irregular. These landings have been associated with oceanographic variation and low primary productivity (8, 30).
Equation 10	cooperation of traders	buyers are able to interact in a reciprocal fashion. Cooperation within the group of traders declines if new traders and new relationships have to be built as a consequence of moving to new areas. We use an exponential functional form to represent rapid decline in cooperation due to increasing trader group size	empirical observations, (10, 31) & group cooperation dynamics (11).	
Equation 11	fishers' price	proportion $M = 1 - R$ of catches sold at theoretical market price and proportion R of catches sold at minimum price. We assume minimum price equals cost of fishing	(7)	average per volume fishers' price is lowest for Santa Rosalìa and the margin between fishers' and export price is highest for Santa Rosalìa, second highest for Guaymas and lowest for the remaining fishery reporting offices (figure S2a). (10) report an increase in fishers' price in Puerto San Carlos (Pacific) in 2010 after the fishery moved into a temporary fishing ground.

References

1. Clark CW (1985) *Bioeconomic modelling and fisheries management.*
2. Mansal F, Nguyen-Huu T, Auger P, Balde M (2014) A mathematical model of a fishery with variable market price: sustainable fishery/over-exploitation. *Acta biotheoretica* 62(3):305–323.
3. Urías-Sotomayor R, et al. (2018) Stock assessment of jumbo squid Dosidicus gigas in northwest Mexico. *Latin american journal of aquatic research* 46(2):330–336.
4. Hoving HJT, et al. (2013) Extreme plasticity in life-history strategy allows a migratory predator (jumbo squid) to cope with a changing climate. *Global change biology* 19(7):2089–2103.
5. Fryxell JM, et al. (2017) Supply and demand drive a critical transition to dysfunctional fisheries. *Proceedings of the National Academy of Sciences* 114(46):12333–12337.
6. De La Cruz González F (2007) Ph.D. thesis.
7. Giovannini A (1988) Exchange rates and traded goods prices. *Journal of international Economics* 24(1-2):45–68.
8. Robinson CJ, Gómez-Gutiérrez J, Markaida U, Gilly WF (2016) Prolonged decline of jumbo squid (Dosidicus gigas) landings in the Gulf of California is associated with chronically low wind stress and decreased chlorophyll a after El Niño 2009–2010. *Fisheries research* 173:128–138.
9. De la Cruz-González J, et al. (2011) Análisis socioeconómico de la pesquería de calamar gigante en Guaymas, Sonora. *Economía, Sociedad y Territorio* 11(37):645–666.
10. Schneller AJ, Lara VCFd, Zavala CAS, Rebollo AM (2015) Socioeconomic diagnosis of the 2010 jumbo squid artisanal fishery near Magdalena Bay, Baja California Sur, Mexico. *Hidrobiológica (México) Num.1 Vol.24*.
11. Casari M, Tagliapietra C (2018) Group size in social-ecological systems. *Proceedings of the National Academy of Sciences* 115(11):2728–2733.
12. Meyer J, Cramon-Taubadel Sv (2004) Asymmetric Price Transmission: A Survey. *Journal of Agricultural Economics* 55(3):581–611.
13. Heckman J (1974) Shadow prices, market wages, and labor supply. *Econometrica: journal of the econometric society* pp. 679–694.
14. Bezanson J, Edelman A, Karpinski S, Shah V (2017) Julia: A Fresh Approach to Numerical Computing. *SIAM Review* 59(1):65–98.
15. Wächter A, Biegler LT (2006) On the implementation of an interior-point filter line-search algorithm for large-scale nonlinear programming. *Mathematical Programming* 106(1):25–57.
16. Dunning I, Huchette J, Lubin M (2017) JuMP: A Modeling Language for Mathematical Optimization. *SIAM Review* 59(2):295–320.
17. Pecl GT, Jackson GD (2008) The potential impacts of climate change on inshore squid: biology, ecology and fisheries. *Reviews in Fish Biology and Fisheries* 18(4):373–385.
18. Robinson CJ, Gómez-Gutiérrez J, de León DAS (2013) Jumbo squid (Dosidicus gigas) landings in the Gulf of California related to remotely sensed SST and concentrations of chlorophyll a (1998–2012). *Fisheries Research* 137:97–103.
19. Field JC, et al. (2013) Foraging ecology and movement patterns of jumbo squid (Dosidicus gigas) in the California Current System. *Deep Sea Research Part II: Topical Studies in Oceanography* 95:37–51.
20. Arkhipkin A, Argüelles J, Shcherbich Z, Yamashiro C (2014) Ambient temperature influences adult size and life span in jumbo squid (Dosidicus gigas). *Canadian Journal of Fisheries and Aquatic Sciences* 72(3):400–409.
21. SAGARPA (2017) Anuario estadistico de pesca 2017, (SAGRAPA- CONAPESCA, ganaderia, desarollo rural, pesca y alimentacion, Secretaria de agricultura), Technical report.
22. Frawley TH, et al. (in review) Impacts of a Transition to Tropical Oceanic Conditions on Gulf of California Jumbo Squid (2010-2015). *ICES Journal Marine Sciences.*
23. INEG Mexico (2019) Indice Nacional de Precios al Consumidor mexico (INPC).
24. Comisión Reguladora de Energía (2018) Historial de precios promedio al público de gas LP reportados por los distribuidores.
25. Mascareñas I (2017) Mexico's National fisheries statistics, 2001-2016.
26. Ibánez CM, et al. (2016) Population dynamics of the squids Dosidicus gigas (Oegopsida: Ommastrephidae) and Doryteuthis gahi (Myopsida: Loliginidae) in northern Peru. *Fisheries Research* 173:151–158.
27. García-Rodríguez JR (1995) Observaciones sobre la pesquería del calamar gigante en el Golfo de California.
28. Gordon HS (1954) The Economic Theory of a Common-Property Resource: The Fishery. *Journal of Political Economy* 62(2):124–142.
29. Burgess MG, et al. (2017) Range contraction enables harvesting to extinction. *Proceedings of the National Academy of Sciences* 114(15):3945–3950.
30. Stewart JS, et al. (2014) Combined climate-and prey-mediated range expansion of Humboldt squid (Dosidicus gigas), a large marine predator in the California Current System. *Global change biology* 20(6):1832–1843.
31. Raya L, et al. (2009) Análisis del consumo de calamar gigante en el noroeste de México. *Región y sociedad* 21(46):145–157.
